# Impact of the SARS-CoV-2 (COVID-19) Pandemic on Characteristics and Management of Uveal Melanoma in the National Referral Center in Poland

**DOI:** 10.3390/cancers16112061

**Published:** 2024-05-29

**Authors:** Bożena Romanowska-Dixon, Michał Szymon Nowak, Janusz Śmigielski, Magdalena Dębicka-Kumela

**Affiliations:** 1 Department of Ophthalmology, Jagiellonian University Collegium Medicum, 38 Kopernika Str., 31-501 Krakow, Poland; romanowskadixonbozena1@gmail.com (B.R.-D.); magda@kumela.pl (M.D.-K.); 2 Ophthalmology and Ophthalmic Oncology Clinic, University Hospital, 38 Kopernika Str., 31-501 Krakow, Poland; 3 Institute of Optics and Optometry, University of Social Science, 121 Gdanska Str., 90-519 Lodz, Poland; 4 Provisus Eye Clinic, 112 Redzinska Str., 42-209 Czestochowa, Poland; 5 Department of Statistics, State University of Applied Science in Konin, 1 Przyjazni Str., 65-510 Konin, Poland; janusz.smigielski.stat@gmail.com

**Keywords:** uveal melanoma, brachytherapy, proton therapy, enucleation

## Abstract

**Simple Summary:**

Severe acute respiratory syndrome coronavirus 2 (SARS-CoV-2)(COVID-19) has been reported in China since December 2019, and the global outbreak began in early 2020, with the first lockdowns in Europe implemented in February 2020. Restrictions taken to limit the exposure of patients to virus contagion had a notable impact for non-COVID-19 pathologies, including eye cancers. Despite the fact that uveal melanoma is the most common primary intraocular cancer in adults, the number of publications on the impact of the COVID-19 pandemic on the characteristics and treatment of uveal melanoma is limited, and most of them have included small patient samples. We studied the impact of the COVID-19 pandemic on the characteristics and management of uveal melanoma (UM) in a group of 1336 patients from the National Referral Center in Poland.

**Abstract:**

(1) Background: to analyze the impact of the COVID-19 pandemic on the characteristics and management of uveal melanoma (UM) in the National Referral Center in Poland. (2) Materials and Methods: the retrospective analysis of 1336 patients who were newly diagnosed with UM at the Department of Ophthalmology and Ophthalmic Oncology, Jagiellonian University Collegium Medicum Krakow, Poland between 1 January 2018 and 31 December 2021. The demographic and clinical data were compiled, including localization, size, and treatment methods of tumors. (3) Results: In total, 728 patients with UM were included before the COVID-19 pandemic, in the years 2018–2019, and 608 were included during the COVID-19 pandemic, in the years 2020–2021. Fixed-base dynamics indicators for the incidence of uveal melanoma (base year 2018) in the National Referral Center in Poland were 80.22% and 86.81% in the years 2020 and 2021, respectively. UMs were statistically significantly larger and more frequently localized anterior to the equator of the eye globe in the year 2021 than in the year 2018 (Chi-square Pearson test *p* = 0.0001 and *p* = 0.0077, respectively). The rate of patients treated with enucleation increased from 15.94% in the year 2018 to 26.90% in the year 2021 (Chi-square Pearson test *p* = 0.0005). (4) Conclusions: Statistically significant differences were found in the management of uveal melanoma in the National Referral Center in Poland during the COVID-19 pandemic with tumors being larger, more frequently localized anterior to the equator of the eye globe, and more often enucleated.

## 1. Introduction

Uveal melanoma (UM) is the most common primary intraocular cancer in adults characterized by high mortality observed in Poland [1]. Our study on the incidence and survival of ocular melanoma in the National Cancer Registry of Poland during 2010–2017 showed that in our country, 8.4% of patients diagnosed with UM died within one year, and 39.2% died within five years from the initial diagnosis, which provides the one-year and five-year mortality rates of 8.4% and 39.2%, respectively [1]. The one-year overall survival (OS) was 91.6%, and the five-year OS was 60.8%. However, our mortality rate was higher than that found in Israel, Singapore, Sweden, Denmark, and the United Kingdom, and it was comparable with data from an epidemiological study of uveal melanoma from the US Surveillance, Epidemiology, and End Results Program from 2010–2015, where the five-year OS was 61.8% [2,3,4,5,6,7]. Previously published studies have shown that more than 50% of patients develop metastases within 15 years of the initial diagnosis and found that older age at diagnosis, severe tumor stage, distant metastasis, and lack of radiation therapy were associated with a higher risk of cancer death [4,6,7,8,9,10,11]. Given the poor prognosis of patients with uveal melanoma, early detection and treatment initiation are crucial for overall survival.

Severe acute respiratory syndrome coronavirus 2 (SARS-CoV-2) has been reported in China since December 2019, and the global outbreak began in early 2020, with the first lockdowns in Europe implemented in February 2020 [12]. Restrictions put in place to prevent the spread of the virus have impacted the diagnosis and treatment of chronic eye diseases, and uveal melanoma is no exemption [13,14,15,16]. Despite the fact that the COVID-19 pandemic has had an unprecedented impact on health care systems around the world, the number of publications on the impact of the COVID-19 pandemic on the characteristics and treatment of uveal melanoma is limited [17,18,19,20], and most of them have included small patient samples. The US study included eighty patients with uveal melanoma and covered the pandemic period from May 2020 to March 2021. The Spanish study included eighty-two patients with uveal melanoma and covered the pandemic period from March 2020 to March 2021. A study conducted in Ireland included 97 patients and compared uveal melanomas diagnosed in 2020 with melanomas diagnosed in 2019. Only the UK study included a large group of patients—a total of nine hundred and twenty-seven patients with a confirmed diagnosis—but it covered only two periods (March–June and July–October) of the first year of the COVID-19 pandemic.

The present study aimed to analyze the impact of the COVID-19 pandemic on the characteristics and management of uveal melanoma (UM) in a large group of patients in the National Referral Center in Poland during 2018–2021.

## 2. Materials and Methods

### 2.1. Data Sources, Patients, and Definitions

Recruitment methods for this study have been described in detail in our previous work [21]. Briefly, the study design was a retrospective case series. The Department of Ophthalmology and Ophthalmic Oncology of the Collegium Medicum of the Jagiellonian University in Krakow is the National Referral Center for adult patients with eye cancer in Poland, where the majority of uveal melanoma patients in Poland are treated. The hospital database contains medical data including diagnoses coded according to the International Classification of Diseases, 10th revision (ICD-10) and the 3rd edition of the International Classification of Diseases for Oncology (ICD-O-3), as well as all procedures performed using the International Classification of Diseases procedure codes, ICD-9 diseases (ICD-9) and unique National Health Fund codes corresponding to specific hospital procedures, as well as demographic characteristics such as PESEL (personal ID), date of birth, patient gender, and place of residence.

All patients newly diagnosed with uveal melanoma and treated between 1 January 2018 and 31 December 2021 were retrieved from the hospital database and included in this study. Demographic and clinical data were analyzed, including the following: patient gender, age at diagnosis, year of diagnosis, laterality of the tumor (right or left eye), intraocular location, and tumor stage according to the TNM classification of malignant tumors (both at the time of diagnosis), as well as treatment methods, including plaque radiotherapy (iodine-125 or rhutenium-106 brachytherapy), proton beam irradiation (PBI), local surgery, and/or ocular enucleation.

### 2.2. Statistical Analyses

Statistical analyses included a standard annual analysis of the incidence of uveal melanoma and an analysis of fixed-base indicators of the dynamics of the incidence of uveal melanoma (base year 2018) in the National Referral Center in Poland in 2018–2021. They also included the analysis of clinical features, i.e., tumor laterality, tumor location (divided also into anterior or posterior to the equator of the eye), tumor stages, and treatment methods. In Poland, the restrictions in people’s movement during the COVID-19 pandemic were implemented in the periods March–May 2020, November 2020–January 2021, and March–April 2021. Because uveal melanoma is a chronic disease, we decided to compare the results from the second year of the pandemic (2021) with the base year (2018), because we believe this better reflects the impact of the COVID-19 pandemic on patients with uveal melanoma. Differences in age distribution were tested using the Student’s *t* test, and other differences were tested using a Chi-square (χ2) test. Commercially available STATISTICA v. 13.0 PL software (StatSoft Polska, Kraków, Poland) was used for all statistical analyses. *p* values < 0.05 were considered statistically significant. Microsoft Excel 2021(Microsoft Corporation, Redmond, Washington, DC, USA) was used to produce the graphs and figures. The study complied with the assumptions of the Helsinki Declaration regarding research involving humans and was approved by the Institutional Review Board of the Jagiellonian University Medical College (informed consent was waived).

## 3. Results

**Figure 1:**
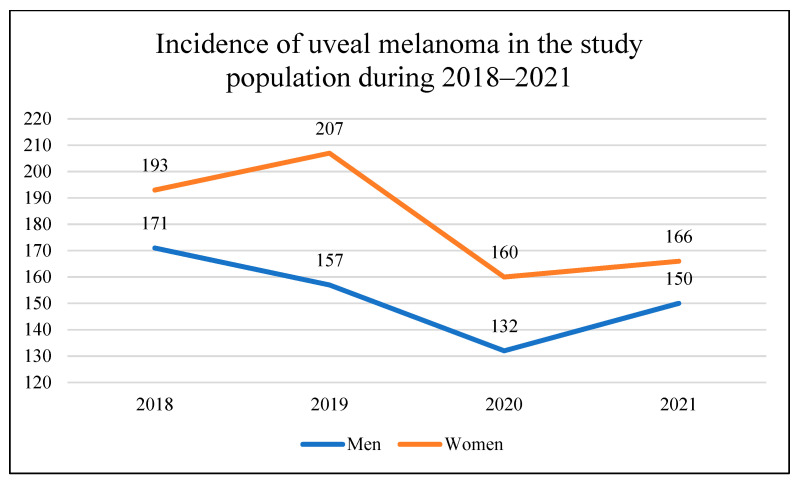
Incidence of uveal melanoma in the National Referral Center in Poland during 2018–2021.

**Figure 2:**
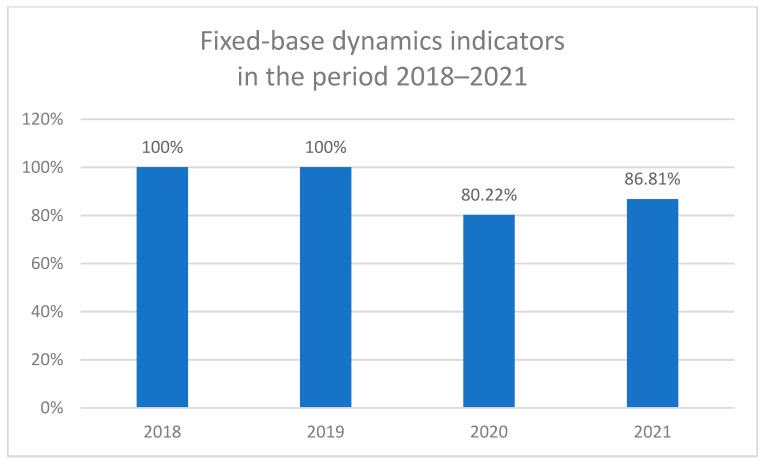
Fixed-base dynamics indicators for the incidence of uveal melanoma (base year 2018) in the National Referral Center in Poland during 2018–2021.

In total, 1336 patients with uveal melanoma (UM) were identified and included in this study in the National Referral Center in Poland between 1 January 2018 and 31 December 2021 (Table 1, Figure 1). Of them, 728 were included before the SARS-CoV-2 (COVID-19) pandemic in the years 2018–2019, and 608 were included during the COVID-19 pandemic in the years 2020–2021. There were 726 women (54.3%) and 610 men (45.7%) in the study population. The sex distribution was similar to that found among patients with uveal melanoma in the National Cancer Registry of Poland (statistical analysis—Chi-square test: χ2 = 0.20, *p* = 0.6570) [1]. The mean age of patients was 63.8 ± 13.8 years, at the time of diagnosis. Fixed-base dynamics indicators for the incidence of uveal melanoma (base year 2018) in the National Referral Center in Poland during 2018–2021 are presented in Figure 2. Comparing to the year 2018, the number of patients with a diagnosis of uveal melanoma decreased during the COVID-19 pandemic to 80.2% and 86.8% in the years 2020 and 2021, respectively. However, we did not find statistically significant differences in the sex distribution and age of patients before and during the COVID-19 pandemic (Table 1).

**Figure 3:**
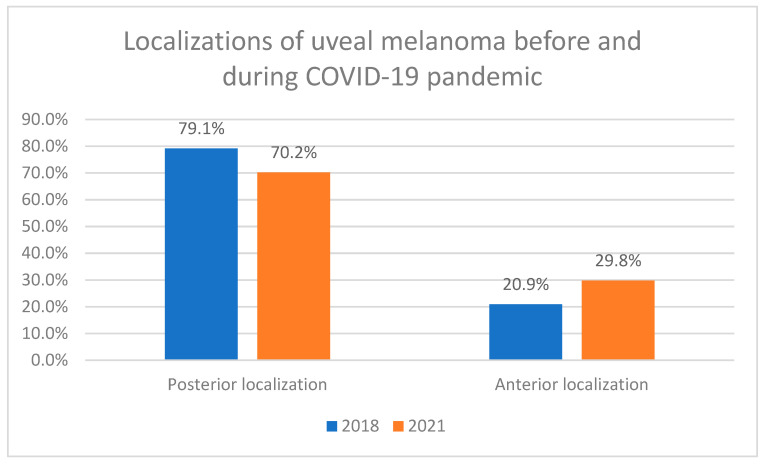
Localizations of uveal melanoma in the National Referral Center in Poland before and during the COVID-19 pandemic. Chi-square Pearson test *p* = 0.0077.

During the study period, 664 uveal melanomas were located in the right eye and 672 in the left eye (49.7% and 50.3%, respectively). The detailed location of uveal melanoma in the National Referral Center in Poland in 2018–2021 is presented in Table 2. A total of 1024 (76.6%) of all UMs were located in the choroid, 151 (11.3%) in the choroid and ciliary body, 49 (3.7%) in the iris, 71 (5.3%) in the iris and ciliary body, 29 (2.2%) in the ciliary body, and 12 (0.9%) in the iris, ciliary body, and choroid, at diagnosis. Statistical analysis showed that during the COVID-19 pandemic, UMs were statistically significantly more often located anterior to the equator of the eyeball than before the COVID-19 pandemic: 29.75% in 2021 vs. 20.88% in 2018 (Chi-square Pearson test *p* = 0.0077) (Figure 3).

In the National Referral Center in Poland, during the study period 2018–2021, 347 (26.0%) of all UMs were classified as T1, 392 (29.3%) as T2, 382 (28.6%) as T3, and 215 (16.1%) as T4, at the time of diagnosis (Table 3). During the COVID-19 pandemic, the number of T4 tumors significantly increased to 26.3% in the year 2021 (from 8.8% in the year 2018), and simultaneously the number of T1 tumors significantly decreased to 30.4% in the year 2021 (from 34.1% in the year 2018) (Chi-square Pearson test *p* = 0.0001).

**Figure 4:**
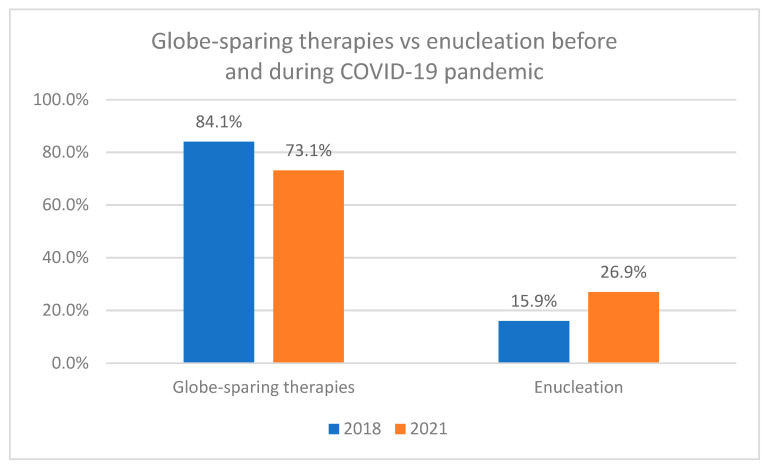
The treatment methods of uveal melanoma in the National Referral Center in Poland before and during the COVID-19 pandemic. Chi-square Pearson test *p* = 0.0005.

The analysis of the medical management of UM in our patients is presented in Table 4. In the study group, 909 (68.0%) of all cancers were treated with plaque brachytherapy, including 405 (30.3%) with iodine-125 and 504 (37.7%) in the case of ruthenium-106, 36 (2.7%) tumors were treated with local surgery combined with plaque brachytherapy (iodine-125 or rhutenium-106), and 117 (8.8%) tumors were treated with proton beam irradiation (PBI). Enucleation was used as the primary treatment in 274 (20.5%) tumors. Statistical analysis revealed that during the COVID-19 pandemic, Ums were statistically significantly more frequently enucleated than before the COVID-19 pandemic: 26.9% in the year 2021 vs. 15.9% in the year 2018 (Chi-square Pearson test *p* = 0.0005) (Figure 4).

## 4. Discussion

The COVID-19 pandemic resulted in unprecedented disruption to healthcare, and restrictions taken to limit the exposure of patients to virus contagion had a notable impact for non-COVID-19 pathologies, including eye cancers [17,18,19,20]. The incidence of uveal melanoma varies among ethnic groups and regions around the world. Most previously published studies have shown a higher incidence of ocular melanoma among men, with a decreasing north–south gradient in the incidence of uveal melanoma occurring in Europe [22,23,24,25,26,27,28]. However, this incidence has remained stable over recent decades [1,8,22]. Our previously published study showed a higher incidence of ocular melanoma among women, and the total incidence of uveal melanoma in the general population of Poland in 2010–2017 was 6.67/1,000,000 person-years. The mean age at the time of uveal melanoma diagnosis in the general population of Poland was 62.7 ± 14.4 years [1]. In the present study, the mean age of patients was 63.8 ± 13.8 years, and we did not find any statistically significant differences in the sex distribution and age of patients before and during the COVID-19 pandemic. Yet, the present study showed that the number of patients (in the National Referral Center in Poland) with a diagnosis of uveal melanoma decreased significantly during the COVID-19 pandemic (to 80.2% and 86.8% in the years 2020 and 2021, respectively), compared to the years 2018–2019. Our results were similar to data from the United Kingdom (UK), which saw a 43.2% reduction in uveal melanoma diagnoses during the first four months of the COVID-19 pandemic (March–June 2020) [18]. However, studies conducted in the US, Ireland, and Spain have found no reduction in the number of patients with newly diagnosed uveal melanoma during the COVID-19 pandemic, but these studies included a small number of patients [17,19,20].

Our study also showed that during the COVID-19 pandemic, uveal melanomas were statistically significantly larger and more frequently localized anterior to the equator of the eye globe. The number of T4 tumors increased from 8.8% in the year 2018 to 26.3% in the year 2021, and the number of uveal melanomas localized anterior to the equator of the eye globe increased from 20.9% in the year 2018 to 29.8% in the year 2021, respectively. Our results were consistent with those from studies conducted in Spain and Ireland, where increased tumor size at diagnosis was found during the COVID-19 pandemic [17,19]. Additionally, the Irish study found that tumors tended to be larger in men both before and during the COVID-19 pandemic, which was consistent with the results of our previous study on sex differences in uveal mealanoma treatment in Poland in 2018–2021 [21]. A study in the UK also found that more patients presented with more advanced cancers post-lockdown [18]. In contrast to these results, a study conducted in Texas (United States of America) showed that the COVID-19 pandemic had no impact on the presentation of patients with uveal melanoma in terms of all tumor characteristics, including size, stage, and gene expression data [20]. The difference in results may be attributed to the regional context of the studies, with Texas having a different approach to the COVID-19 pandemic: the Texas government has never issued an isolation order [20]. However, the genetic findings from Texas were consistent with an Irish study in which mutations in the BRCA1-associated protein 1 (BAP1) gene were identified in 14.3% of people who underwent genetic analysis and found no significant differences in the genetics or histology of uveal tumors before the COVID-19 pandemic or during it. Although studies from other countries did not analyze the specific localization of uveal melanomas, researchers from Spain and Ireland confirmed a greater number of patients diagnosed with extraocular extension of tumors compared to the pre-pandemic era [17,19].

The current eye cancer treatment strategy aims to save the patient’s life, vision, and cosmetics, according to priority. Treatment depends on the location of the tumor, its size, local extent, visual acuity, and systemic condition. Most patients with ocular melanoma are currently treated with global sparring methods, including plaque brachytherapy, laser photocoagulation, transpupillary thermotherapy, particle beam radiotherapy, gamma knife radiosurgery, and local surgical resection [1,8,9,10,11,29]. Contrary to this trend, our study showed that during the COVID-19 pandemic, the number of enucleations increased significantly from 15.9% in the year 2018 to 26.9% in the year 2021. Our results were consistent with studies conducted in Spain and Ireland, where the number of enucleations increased significantly, from 11.9% and 9.3%, respectively, before the COVID-19 pandemic, to 47.5% and 21.6%, respectively, during the COVID-19 pandemic [17,19]. A study conducted in Spain also showed that patients diagnosed during the pandemic had a statistically significantly increased risk of treatment with the enucleation method. A study in the UK also found an increased number of enucleations among patients diagnosed during the first lockdown (March–June 2020). However, they believe that there was no conscious intention to favor enucleation over globe-sparing therapies in the UK or elsewhere, and this increase was due to a trend towards reducing the risk of transmitting the virus during isolation [20,30,31]. The largest decline in the number of globe-sparing therapies, in the National Referral Center in Poland, was observed in the first year of the COVID-19 pandemic in proton beam radiotherapy, which is consistent with the studies from the United Kingdom and Ireland [18,19]. Proton beam radiotherapy requires multiple hospitalizations, which was difficult during strict lockdowns in the first year of the pandemic and could also increase the risk of contracting the virus. Another noteworthy fact is that in 2021 the total number of globe-sparing therapies in Poland was lower compared to 2020, due to the increase in tumor size observed in uveal melanoma patients during the COVID-19 pandemic.

One major limitation of our study is the lack of survival analysis of patients with uveal melanoma. However, we believe that the follow-up period was influenced by the COVID-19 pandemic, which may have influenced the cause of death in some patients; the large population size is the major strength of the present study.

In summary, to the best of our knowledge, this is the largest study on the impact of the COVID-19 pandemic on the characteristics and treatment of uveal melanoma. Advice on staying at home, while reducing the risk of contracting the virus, resulted in the increase in tumor size seen in uveal melanoma patients during the COVID-19 pandemic. It could also cause delays in cancer diagnosis and treatment, which could negatively impact patient survival. However, our results are specific only to Poland and cannot describe other healthcare systems. Still, the impact of the COVID-19 pandemic is not exclusive to uveal melanoma. Studies from the Netherlands and Australia showed a significant decrease in the number of cancer diagnoses—by 27% and 10%, respectively, during the COVID-19 restrictions. Other studies in the UK and US have found increased mortality from various cancers during the COVID-19 pandemic due to the deferral of interventions such as surgery and on-site cancer care [32,33,34]. There is also a potential risk that large numbers of patients with uveal melanoma and other cancers will remain undiagnosed in the community, and it is likely that there will be a sharp increase in cancer cases once the COVID-19 pandemic ends [17,18,19,20].

## 5. Conclusions

Statistically significant differences were found in the characteristics and management of uveal melanoma in the National Referral Center in Poland during the COVID-19 pandemic, with tumors being larger, more frequently localized anterior to the equator of the eye globe, and more often enucleated. The present study also showed that the number of patients with a diagnosis of uveal melanoma decreased significantly during the COVID-19 pandemic, when compared to the pre-pandemic era.

## Figures and Tables

**Table 1:** Demographic analysis of patients with uveal melanoma in the National Referral Center in Poland before and during the COVID-19 pandemic.

Time Period	2018–2019			2020–2021		
	n %	Age, Mean	Age, Median	n %	Age, Mean	Age, Median
Men	328 (45.1%)	63.4 ± 13.4	65.0	282 (46.4%)	63.3 ± 13.2	65.0
Women	400 (54.9%)	65.1 ± 13.6	66.0	326 (53.6%)	63.3 ± 15.2	65.0
All	728 (100%)	64.3 ± 13.5	66.0	608 (100%)	63.3 ± 14.7	65.0

Sex distribution: Chi-square Pearson test *p* = 0.6278; patients’ age: Student’s *t* test *p* = 0.1942.

**Table 2:** Localizations of uveal melanoma in the National Referral Center in Poland during 2018–2021.

Localization of Tumor	Year				All n (%)
	2018 n (%)	2019 n (%)	2020 n (%)	2021 n (%)	
Choroid	288 (79.1%)	278 (76.4%)	236 (80.8%)	222 (70.2%)	1024 (76.6%)
Choroid and ciliary body	28 (7.7%)	41 (11.3%)	28 (9.6%)	54 (17.1%)	151 (11.3%)
Iris	17 (4.7%)	12 (3.3%)	6 (2.1%)	14 (4.4%)	49 (3.7%)
Iris and ciliary body	20 (5.5%)	23 (6.3%)	10 (3.4%)	18 (5.7%)	71 (5.3%)
Ciliary body	7 (1.9%)	7 (1.9%)	11 (3.8%)	4 (1.3%)	29 (2.2%)
Iris, ciliary body, and choroid	4 (1.1%)	3 (0.8%)	1 (0.3%)	4 (1.3%)	12 (0.9%)
All	364 (100%)	364 (100%)	292 (100%)	316 (100%)	1336 (100%)

**Table 3:** Cancer stages according to TNM classification of malignant tumors (at the time of diagnosis) in the National Referral Center in Poland during 2018–2021.

Cancer Stage TNM	Year				All n (%)
	2018 n (%)	2019 n (%)	2020 n (%)	2021 n (%)	
T1	124 (34.1%)	106 (29.1%)	66 (22.6%)	96 (30.4%)	392 (29.3%)
T2	109 (29.9%)	114 (31.3%)	88 (30.1%)	71 (22.4%)	382 (28.6%)
T3	99 (27.2%)	94 (25.8%)	88 (30.1%)	66 (20.9%)	347 (26.0%)
T4	32 (8.8%)	50 (13.8%)	50 (17.2%)	83 (26.3%)	215 (16.1%)
All	364 (100%)	364 (100%)	292 (100%)	316 (100%)	1336 (100%)

Chi-square Pearson test *p* = 0.0001.

**Table 4:** The treatment methods of uveal melanoma in the National Referral Center in Poland during 2018–2021.

Treatment Methods	Year				n (%)
	2018 n (%)	2019 n (%)	2020 n (%)	2021 n (%)	
Plaque brachytherapy with iodine-125	125 (34.4%)	103 (28.3%)	103 (35.3%)	74 (23.4%)	405 (30.3%)
Plaque brachytherapy with rhutenium-106	137 (37.6%)	134 (36.8%)	116 (39.7%)	117 (37.0%)	504 (37.7%)
Local surgery with plaque brachytherapy	7 (1.9%)	11 (3.0%)	6 (2.0%)	12 (3.8%)	36 (2.7%)
Proton beam irradiation (PBI)	37 (10.2%)	38 (10.5%)	14 (4.8%)	28 (8.9%)	117 (8.8%
Enucleation	58 (15.9%)	78 (21.4%)	53 (18.2%)	85 (26.9%)	274 (20.5%)
All	364 (100%)	364 (100%)	292 (100%)	316 (100%)	1336 (100%)

## Data Availability

Data are contained within the article.
